# Managing Emotional Intensity Difficulties in Older Adults With Personality Disorders: A Proof‐of‐Concept Study

**DOI:** 10.1002/jclp.70090

**Published:** 2026-01-09

**Authors:** Erol Ekiz, Sebastiaan P. J. van Alphen, Machteld A. Ouwens, Arjan C. Videler

**Affiliations:** ^1^ PersonaCura, Clinical Center of Excellence for Personality and Developmental Disorders in Older Adults GGz Breburg Tilburg the Netherlands; ^2^ Tranzo, Scientific Centre for Care and Wellbeing of the Tilburg School of Social and Behavioral Sciences of Tilburg University Tilburg the Netherlands; ^3^ Clinical Center of Excellence for Personality Disorders in Older Adults Mondriaan Mental Health Center Heerlen‐Maastricht the Netherlands; ^4^ Department of Psychology (PE), Personality and Psychopathology Research Group (PEPS) Vrije Universiteit Brussel (VUB) Brussels Belgium; ^5^ Department of Clinical Psychological Science, Faculty of Psychology and Neuroscience Maastricht University Maastricht the Netherlands

**Keywords:** borderline personality disorder, emotional dysregulation, older adults, personality disorder, psychotherapy

## Abstract

Problematic emotion management is a core symptom of personality disorders and does not tend to improve spontaneously with age. Systems Training for Emotional Predictability and Problem Solving (STEPPS), a treatment program targeting emotional intensity difficulties, has been found to be effective for younger adults with borderline personality disorder. After a pilot study and a Delphi study, STEPPS was adjusted for older adults to better suit this population. The aim of present study was to evaluate first outcomes (e.g., level of improvement) of the adjusted STEPPS Older Adults (STEPPS‐OA). A total of 52 patients, with a mean age of 67 years (range: 60–80), participated in this proof‐of‐concept study with pre‐, mid‐, and post‐treatment measurement points. A total number of 38 patients completed the treatment; 14 patients (27%) dropped out. A majority of the patients (58%) had borderline personality disorder. After treatment, patients reported significantly decreased borderline personality disorder severity and symptomatic distress. Furthermore, there was a significant improvement of adaptive emotion regulation strategies and all personality functioning factors. Finally, some maladaptive personality traits (i.e., Disinhibition and Negative Affectivity) decreased significantly. Results of this proof‐of‐concept study indicate STEPPS‐OA is a promising treatment option for managing emotional intensity difficulties for older adults with personality disorders.

## 1 Introduction

In Lev Tolstoy's classic, the young protagonist Anna Karenina struggles with intense emotions and turbulent relationships. Her inability to manage her emotions leads her into a series of emotional outbursts and impulsive decisions, ultimately resulting in her social isolation and tragic end. One wonders, what might her life have looked like as Anna Karenina would have aged and grown old with her tumultuous and turbulent feelings? Is emotional dysregulation only a challenge for younger adults, or does it also affect older adults, perhaps in ways that may present differently, complicating recognition by professionals? Despite the clinical relevance, evidence based treatment for emotional intensity difficulties in older adults remain scarce. Against this background, the present study explores whether emotion regulation skills can still be acquired later in life.

Emotion regulation can be described as the process of shaping which emotions one feels, when one feels them, and how one expresses them, encompassing changes in the onset, intensity, length, and interplay of emotional responses in terms of behavior, experience, and physiology (Gross 2002). Emotion regulation has a key role in the development of psychiatric conditions like borderline personality disorder (BPD) (Putnam and Silk 2005). Especially in the nineties, various psychotherapeutic treatments, targeting the emotion regulation skills of patients with BPD, were developed and implemented (Linehan 1993; Bartels and Crotty 1998; Blum et al. 2008). Systems Training for Emotional Predictability and Problem Solving (STEPPS) is one of those treatments, originally developed to reduce hospitalization rates, self‐harm acts and other destructive behaviors in patients with BPD (Blum et al. 2002). Reframing BPD as Emotional Intensity Disorder, STEPPS targets patients with emotional intensity difficulties in particular (Blum et al. 2008). Emotional intensity difficulties seem to occur when biological, psychological, and social factors have led to certain emotional sensitivities. People with emotional intensity difficulties show heightened sensitivity to emotional stimuli, respond more intensely (internally and/or externally) to emotional triggers, and require more time to calm down, leading to a greater likelihood of emotions piling up (Blum et al. 2016). STEPPS is a manualized group therapy program, consisting of 20 weekly meetings. It draws from Cognitive Behavioral Therapy (CBT), emphasizing the practice of new behaviors and awareness of the interplay between thoughts, emotions and actions. The school‐like approach, which includes homework assignments and focused discussions, avoids delving into participants’ individual issues and past traumas, concentrating instead on building skills to manage emotional fluctuations. The group program consists of three major components. First, patients receive psychoeducation about emotional intensity difficulties. Second, patients are taught five emotion management skills. In this part, patients learn and practice (1) distancing themselves from emotions and thoughts, in order to observe what is there without judgment, (2) communicating what is experienced, felt and thought, (3) detecting and challenging thought distortions, (4) shifting attention from negative to neutral or positive stimuli, and (5) managing problems by learning to tackle them in a structured manner. Finally, during the third component, patients are taught healthy lifestyle behaviors and interpersonal effectiveness. In short, the emotion management skills help patients manage rising emotions when they occur. The behavior management skills enable patients to structure their lives in a way that reduces the occurrence of rising emotions. Noteworthy, the patient's support group plays an active role in the treatment, for instance, through support group meetings. Members of this group support and reinforce the patient in the use of their newly learned skills.

A recent systematic review showed that STEPPS is a promising treatment for BPD (Ekiz et al. 2022). STEPPS has a positive effect on symptom severity of BPD, quality of life, symptoms of depression, and negative affectivity. These effects, however, were found in younger adults. Mean age of study participants was 33 years. At the time of the review, there were no studies conducted in older adults. Nonetheless, personality disorders (PDs) are highly prevalent in later life (Penders et al. 2020), as PDs tend to be chronic, might even have a late onset or re‐emerge in late life (Videler et al. 2019). Large‐scale prevalence studies utilizing data from the National Epidemiologic Survey on Alcohol and Related Conditions (NESARC) have found that 10.7%–14.5% of older adults have at least one personality disorder (Holzer and Huang 2019; Reynolds et al. 2015). With respect to BPD, there are indications that BPD does not improve in older adulthood (Tracie Shea et al. 2009). In fact, psychosocial functioning and functional impairment seem to get worse in old age (Tracie Shea et al. 2009; Frías et al. 2017). Furthermore, older adults seem to inhibit and experience emotions similar to younger adults (Malatesta and Kalnok 1984). However, in comparison with younger adults, older adults report that at their age, feelings of anger, sadness and happiness should be more concealed. These findings indicate the ongoing presence of BPD in older adults, likely characterized by more covert or concealed emotional intensity difficulties. This highlights the importance of treatment options for older adults with emotional intensity difficulties.

In a pilot study conducted in 2023, STEPPS was examined as a potential treatment option for older adults with PD (Ekiz et al. 2023a). In this study, older adults (mean age of 63.9 years) reported decreased BPD‐symptoms (large effect size [ES], improved self‐control [large ES], and identity integration [medium ES]), after the STEPPS program. However, older adults did not report significant changes in psychological distress and interpersonal functioning. Since older adults may face additional hurdles in later life, like social isolation, health concerns, and limited support (Cacioppo and Cacioppo 2014; Cornwell and Waite 2009), it is important to find out why these social and psychological health variables did not improve. An important hypothesis was that the original STEPPS program was insufficiently attuned to the lived experience of older adults (Ekiz et al. 2023a). In order to explore this hypothesis, a Delphi study was conducted (Ekiz et al. 2023b). In this study, experts reached consensus on three statements, indicating that the original STEPPS protocol is not sufficiently applicable for older adults with emotional intensity difficulties and PD, and that the program needs adjustments on content and process level for older adults. These proposed adjustments have resulted in a modified STEPPS for older adults (STEPPS‐OA). The current research aims to evaluate the first outcomes of STEPPS‐OA in older adults with PD as a proof‐of‐concept.

## 2 Methods

### 2.1 Study Design

This study was designed as a proof‐of‐concept study. A proof‐of‐concept study serves as an initial assessment of the feasibility and acceptability of a new intervention, often using surrogate markers to evaluate the impact of the treatment (Thabane et al. 2010). It functions as an early‐stage exploration to determine the potential of the concept before embarking on larger scale studies. To investigate the potential of STEPPS‐OA, a naturalistic pre‐, mid‐, and post‐treatment multicenter study was conducted. Parameters of interest were attrition rates and reasons, and level of improvement in BPD‐severity, psychological distress, emotion regulation strategies, personality functioning and personality traits.

### 2.2 Procedure

The original STEPPS protocol was adjusted based on recommendations from experts in the Delphi study (Ekiz et al. 2023b), literature on CBT modifications for older adults (Sorocco and Lauderdale 2011), and feedback from older patients who had been treated with the original STEPPS. We presented the adjusted STEPPS‐OA to some of the experts and to STEPPS practitioners of two mental health clinics in the Netherlands. Following their feedback, additional adjustments were made.

Two out‐patient clinical centers of excellence for PD in older adults and four out‐patient mental health clinics in the Netherlands participated in this study. The study was approved by Medical Ethics Committee Zuyderland (METCZ20190161) and all research ethics committees of the six participating sites. The study was registered in the Overview of Medical Research in the Netherlands (OMON) database (NL‐OMON23728). In participating health centers, older adults with emotional intensity difficulties were referred to a STEPPS practitioner. In one or two intake appointments, patients were given oral and written information about STEPPS‐OA and the current study. Eligible patients were then seen for additional testing to determine inclusion or exclusion for present study. Patients meeting inclusion criteria were asked for their informed consent. Inclusion criteria were: (1) participant is at least 60 years old, (2) participant is diagnosed with a PD according to the DSM‐5 criteria (using the Structured Clinical Interview for DSM‐5 Disorders, section Personality Disorders [SCID‐5‐PD] [First et al. 2016]), (3) participant is diagnosed with a minimum of three BPD symptoms (due to age‐related differences in the expression of BPD symptoms [McMahon et al. 2019]), (4) participant experiences emotional intensity difficulties, (5) participant has (some degree of) willingness to change his/her behavior, and (6) participant is able and willing to create a support group. Inclusion criterion 1 was checked by EE, inclusion criteria 2, 3, and 4 were formally assessed by a psychologist or nurse practitioner of the participating center, and inclusion criteria 5 and 6 were checked by the STEPPS practitioner. EE conducted seven SCID‐5‐P interviews; the remaining interviews were conducted by clinicians who were not part of the research team. Exclusion criteria were: (1) severe cognitive impairments (2) a schizoid, schizotypal or paranoid PD, (3) substance abuse needing clinical detoxification, (4) acute co‐morbid psychiatric conditions like psychosis, bipolar disorder or major depression, (5) learning disabilities (IQ < 80); (6) patients with significant hearing or vision problems to such an extent that they cannot participate in a group. Exclusion criteria 1, 2, and 5 were checked by a psychologist or nurse practitioner of the participating center, exclusion criteria 3, 4, and 6 were checked by the referring therapist and STEPPS practitioner. For criterion 1, the Montreal Cognitive Assessment (MoCA) (Nasreddine et al. 2005) was used as a screening tool, and, if the results indicated potential cognitive impairment, a more comprehensive neuropsychological assessment was conducted. When signs of learning difficulties were present (criterion 5), an additional IQ assessment with the use of the Wechsler Adult Intelligence Scale ‐ Fourth Edition (WAIS‐IV) was conducted.

The group sessions of STEPPS‐OA were led by STEPPS practitioners. These mental health care professionals included psychologists, psychiatric nurses and nurse practitioners, all trained in the STEPPS program. The psychological assessments (i.e., SCID‐5‐PD, MoCA, WAIS‐IV) for the inclusion and exclusion criteria were conducted by either the STEPPS practitioners or (preferably) by independent mental health professionals, depending on availability. To support treatment fidelity and provide a space to discuss questions and challenging situations, online supervision sessions were held for STEPPS practitioners every 3–4 months.

### 2.3 Treatment Protocol

Main adjustments in STEPPS‐OA include incorporating themes related to older age into the program (e.g., mourning, changing of social roles, life review), pacing the learning of certain skills, providing repetition of program content (e.g., providing an abstract at the end of each group session), providing reflection moments on the group sessions, and emphasis throughout the program on positive experiences and positive psychology. As a result of the adjustments, STEPPS‐OA consists of 21 weekly group‐meetings, in contrast to the usual 20 group sessions of the original STEPPS and the usual 19 group sessions of the Dutch STEPPS (Bos et al. 2010). Furthermore, similar to the Dutch version of STEPPS, which was used in the pilot of 2023 in older adults (Ekiz et al. 2023a), patients attended individual sessions every 2 weeks to support commitment to therapy and reinforce newly acquired skills. Finally, two group meetings for the patient's support system were held during the program.

### 2.4 Measures

To determine inclusion and exclusion criteria all patients were tested prior to their participation, using SCID‐5‐PD and MoCA. SCID‐5‐PD assesses the presence of a DSM‐5 PD. The predecessor of the SCID‐5‐PD, known as the SCID‐II, has an excellent face validity (Van Alphen et al. 2023) and excellent inter‐rater reliability (Lobbestael et al. 2011). MoCA was used to assess the level of cognitive impairment in relation to normal cognitive ageing. MoCA has demonstrated high test‐retest reliability and good internal consistency (Nasreddine et al. 2005). A meta‐analytic evaluation of validation studies revealed an optimal cutoff score of 23/30, optimally balancing sensitivity and specificity and providing the highest classification accuracy (Carson et al. 2018). Therefore a cutoff score of 23 was used.

The outcome measures were assessed using the following questionnaires: Borderline Evaluation of Severity over Time (BEST) (Pfohl et al. 2009), Brief Symptom Inventory (BSI) (Derogatis 1975), *Fragebogen zur Erhebung der Emotionsregulation bei Erwachsenen* (FEEL‐E) (Punt 2015), Severity Indices of Personality Problems – Short Form (SIPP‐SF) (Verheul et al. 2008), and Personality Inventory for DSM‐5‐Brief Form (PID‐5‐BF) (Krueger et al. 2012).

As part of the STEPPS group sessions, the BEST was filled in by participants at the start of every group session. The BEST yields an impression of the participant's current status in severity of BPD criteria on a scale of 15 items. The total score can range between 12 (i.e., minimal BPD severity) and 72 (i.e., maximal BPD severity). In a psychometric study, the scale has been found to have a good test‐retest reliability, and internal consistency (i.e., Cronbach's *α* was 0.86 for subjects with BPD) and discriminant validity are excellent (Pfohl et al. 2009).

Symptomatic distress is measured by the symptom checklist BSI. BSI is a self‐report questionnaire consisting of 53 items. It provides an overview of a patient's symptoms and their intensity using nine symptom scales. The reliability of the BSI scales is good and the convergent and divergent validity have been found to be satisfactory, including for older adults (De Beurs 2006). The internal consistency of the BSI total score is excellent, with a Cronbach's *α* of 0.96 (De Beurs and Zitman 2006).

Emotion regulation strategies are assessed using the 72‐item FEEL‐E questionnaire. It addresses emotions such as anger, anxiety, and sadness, each evaluated by the same 24 items. Participants rate the applicability of each item on a five‐point scale. FEEL‐E categorizes 12 emotion regulation strategies, which include both adaptive and maladaptive approaches. FEEL‐E has demonstrated sufficient to satisfactory internal consistence (mean Cronbach's *α* of 0.79), reliability and validity for adults, with normative data available for individuals aged 18–65 (Punt 2015).

The SIPP‐SF is a relatively age‐neutral self‐report questionnaire (Debast et al. 2018) that measures the core components of (mal)adaptive personality functioning, and provides indices of the severity of personality pathology. The SIPP‐SF consists of 60 items and measures five factors (i.e., Identity Integration, Relational Capacities, Responsibility, Self‐Control, Social Concordance). Studies have showed good validity and reliability (internal consistency ranging from Cronbach's *α* 0.81 to 0.88) for the SIPP‐SF in older adults (Rossi et al. 2017; Van Reijswoud et al. 2021).

The PID‐5‐BF is a 25‐item self‐report questionnaire designed to measure maladaptive personality traits. These traits include Antagonism, Detachment, Disinhibition, Negative Affectivity and Psychoticism. Each item is rated on a four‐point Likert scale, ranging from “very false or often false” to “often true or very true.” The PID‐5‐BF demonstrates strong concurrent and criterion validity in older adults, however has a modest face validity and poor to good (Cronbach's *α* ranging from 0.58 to 0.83) internal consistency indices for subscales (Debast et al. 2018; Hyatt et al. 2020).

### 2.5 Statistical Analyses

This study includes three measurement points. Patients received electronic or printed questionnaires at the first group meeting (T1), at the 11th group meeting (T2) and at the 21st group meeting (T3). T1 contains the followings measures: BSI, FEEL‐E, SIPP‐SF, PID‐5‐BF, and the mean score of the weekly BEST in week 1 and 2. T2 contains BSI, FEEL‐E, SIPP‐SF, and the mean score of the BEST in week 10 and 11. T3 contains BSI, FEEL‐E, SIPP‐SF, PID‐5‐BF, and the mean score of the BEST in week 20 and 21.

BSI, FEEL‐E, and SIPP‐SF were compared using repeated measures ANOVA. Subscales of PID‐5‐BF were compared using paired samples *t*‐tests for normally distributed subscales and Wilcoxon signed rank test for not normally distributed subscales. ES was computed using partial eta squared for repeated measures ANOVA (0.01 = small, 0.06 = medium, 0.14 = large) Cohen's *d* for paired samples *t*‐tests (0.20 = small, 0.50 = medium, 0.80 = large), and *r* for Wilcoxon signed rank tests (0.10 = small, 0.30 = medium, 0.50 = large) (Cohen 1988). Underlying assumptions were checked. To assess clinically meaningful change between T1 and T3, the reliable change index (RCI) described by Jacobson and Truax (Jacobson and Truax 1991) was calculated.

The required number of participants was calculated using G*Power 3 (Faul et al. 2007). The following parameters were entered: F‐test, ANOVA: Repeated measures, within factors, two‐tailed, ES F 0.25, alpha 0.05, power 0.9. According to this calculation, the total sample size should be 36.

## 3 Results

### 3.1 Patient Characteristics

A total of 52 participants were included in this study, of whom 14 participants (26.9%) did not complete the treatment. Patient characteristics are presented in Table 1. Reasons for non‐completion included: significant somatic health issues affecting the participant (*N* = 5) or participant's partner (*N* = 1), dissatisfaction with treatment (*N* = 3; e.g., excessive homework), onset of an acute co‐morbid psychiatric condition during treatment (*N* = 1), failure to adhere to group rules (*N* = 1), participant felt improvement early in the treatment and did not complete the program (*N* = 1), premature termination of the group sessions due to attrition of other group members (*N* = 1), and unknown reasons (*N* = 1). There were no significant differences in age (t(50) = 1.301, *p* = 0.199), gender (χ^2^(1) = 0.130, *p* = 0.718), marital status (χ^2^(2) = 0.798, *p* = 0.671), educational level (χ^2^(2) = 0.299, *p* = 0.861) or DSM‐5‐PD classification (χ^2^(3) = 0.914, *p* = 0.822) between participants who completed the treatment and participants who dropped out.

**Table 1:** Patient characteristics.

Characteristic	Completers (*n* = 38)	Drop‐outs (*n* = 14)	Total (*n* = 52)
Age, mean (SD) in years	66.2 (5.6)	68.6 (6.3)	66.9 (5.8)
Age, range	60–80	61–78	60–80
Gender, *n* (%) female			
Female, *n* (%)	29 (76.3%)	10 (71.4%)	39 (75.0%)
Male, *n* (%)	9 (23.7%)	4 (28.6%)	13 (25.0%)
Marital status			
Single/divorced, *n* (%)	17 (44.7%)	7 (50.0%)	24 (46.2%)
Married/living together, *n* (%)	19 (50.0%)	7 (50.0%)	26 (50.0%)
Long distance relationship, *n* (%)	2 (5.3%)	0 (0.0%)	2 (3.8%)
Educational level			
Low educational level, *n* (%)	18 (47.4%)	6 (42.9%)	24 (46.2%)
Middle educational level, *n* (%)	12 (31.6%)	5 (35.7%)	17 (32.7%)
High educational level, *n* (%)	8 (21.1%)	2 (14.3%)	10 (19.2%)
Unknown/missing	0 (0.0%)	1 (7.1%)	1 (1.9%)
DSM‐5‐PD classification			
Borderline PD, *n* (%)	22 (57.9%)	8 (57.1%)	30 (57.7%)
Obsessive–compulsive PD, *n* (%)	1 (2.6%)	0 (0.0%)	1 (1.9%)
Avoidant PD, *n* (%)	1 (2.6%)	1 (7.1%)	2 (3.8%)
Other specified PD, *n* (%)	14 (36.8%)	5 (35.7%)	19 (36.5%)

Abbreviations: high educational level = high level secondary education or higher, low education level = low‐level secondary education or lower, middle educational level = average‐level secondary education, PD = personality disorder.

### 3.2 Outcome Differences

The results of the outcome analyses are summarized in Table 2. Repeated measures ANOVA were conducted to compare the within‐subject means of BEST, BSI, FEEL‐E, and SIPP‐SF. Mauchly's test revealed violations of sphericity for BEST (χ²(2) = 6.377, *p* = 0.041) and SIPP‐SF‐Identity integration (χ²(2) = 10.41, *p* = 0.005), necessitating Greenhouse‐Geisser corrections for this measure and subscale. Non‐significant results were found for Mauchly's test of sphericity for the other questionnaires and subscales. Significant main effects of time were found for most outcome measures. After treatment, participants reported diminished BPD‐severity on the BEST (*F*(1.67, 50.108) = 11.362, *p* < 0.001) and symptomatic distress as measured by the BSI (*F*(2, 74) = 4.664, *p* = 0.012). Moreover, participants reported significant improvement in adaptive emotion regulation strategies measured with the FEEL‐E (*F*(2, 72) = 7.289, *p* = 0.001), and in personality functioning (SIPP‐SF), more specific in improved ability to sense a coherence of identity (*F*(1.583, 55.389) = 7.270, *p* = 0.003), improved ability to engage in intimate and caring relationships (*F*(2, 70) = 7.019, *p* = 0.002), improved ability to set realistic goals and achieve those goals (*F*(2, 68) = 5.882, *p* = 0.004), improved ability to control emotions and impulses (*F*(2, 70) = 17.441, *p* < 0.001), and improved ability to cooperate with others (*F*(2, 70) = 4.132, *p* = 0.020). The significant results yielded medium to large ES. No significant main effect of time was found for maladaptive emotion regulation strategies as measured by the FEEL‐E (*F*(2, 72) = 2.074, *p* = 0.133). Post‐hoc pairwise comparisons, using Bonferroni correction, revealed significant results for all comparisons between T1 and T3, except for maladaptive emotion regulation strategies. None of the comparisons between T2 and T3 reached the significance level.

**Table 2:** Pre‐, mid‐ to post‐treatment comparisons of outcome measures.

Outcome measures	T1 mean (SD)	T2 mean (SD)	T3 mean (SD)	Effect size	*p*	*p* (T1 vs. T2/T2 vs. T3/T1 vs. T3)	RCI (T1 vs. T3)^b^
BEST	33.68 (10.04)	28.84 (9.30)	26.11 (11.34)	0.275 η_p_^2^ (large)	**0.000** a	**0.005** a/0.208/**0.001** a	41.9%/54.8%/3.2%
BSI	1.47 (0.65)	1.35 (0.72)	1.15 (0.72)	0.112 η_p_^2^ (medium)	**0.012** a	0.744/0.131/**0.030** a	57.9%/26.3%/15.8%
FEEL‐E Adaptive strategies	108.84 (20.45)	115.92 (20.48)	118.78 (22.70)	0.168 η_p_^2^ (large)	**0.001** a	**0.024** a/0.754/**0.007** a	16.2%/81.1%/2.7%
FEEL‐E Maladaptive strategies	107.43 (18.43)	108.89 (21.42)	104.59 (19.73)	0.054 η_p_^2^	0.133	1.000/0.059/0.676	5.4%/89.2%/5.4%
SIPP‐SF Identity Integration	27.83 (7.57)	30.36 (7.64)	31.86 (8.18)	0.172 η_p_^2^ (large)	**0.003** a	**0.047** a/0.261/**0.012** a	16.7%/77.8%/5.6%
SIPP‐SF Relational Capacities	29.14 (8.52)	31.44 (8.67)	32.08 (8.54)	0.167 η_p_^2^ (large)	**0.002** a	**0.016** a/1.000/**0.007** a	11.1%/88.9%/0%
SIPP‐SF Responsibility	37.97 (6.80)	39.69 (7.05)	40.71 (7.21)	0.147 η_p_^2^ (large)	**0.004** a	0.058/0.541/**0.020** a	22.9%/74.3%/2.9%
SIPP‐SF Self‐control	29.94 (8.91)	33.36 (8.81)	35.58 (8.12)	0.333 η_p_^2^ (large)	**0.000** a	**0.001** a/0.064/**0.000** ^a^	33.3%/66.7%/0%
SIPP‐SF Social Concordance	35.31 (9.35)	36.86 (7.91)	37.31 (8.39)	0.106 η_p_^2^ (medium)	**0.020** a	0.205/1.000/**0.035** a	2.8%/97.2%/0%
PID‐5‐BF Total	1.08 (0.43)	n/a	0.89 (0.41)	0.560 Cohen's *d* (medium)	**0.002** a	n/a	11.1%/86.1%/2.8%
PID‐5‐BF Antagonism	0.67 (0.53)	n/a	0.57 (0.46)	0.162 *r*	0.333	n/a	2.8%/94.4%/2.8%
PID‐5‐BF Detachment	1.10 (0.72)	n/a	0.93 (0.66)	0.323 Cohen's *d*	0.060	n/a	8.3%/88.9%/2.8%
PID‐5‐BF Disinhibition	0.79 (0.63)	n/a	0.62 (0.60)	0.388 *r* (medium)	**0.020** a	n/a	8.3%/88.9%/2.8%
PID‐5‐BF Negative Affectivity	1.93 (0.60)	n/a	1.62 (0.70)	0.546 Cohen's *d* (medium)	**0.002** a	n/a	25.0%/72.2%/2.8%
PID‐5‐BF Psychoticism	0.91 (0.67)	n/a	0.71 (0.51)	0.331 Cohen's *d*	0.055	n/a	13.9%/83.3%/2.8%

Abbreviations: BEST= Borderline Evaluation of Severity over Time, BSI= Brief Symptom Inventory, FEEL‐E= *Fragebogen zur Erhebung der Emotionsregulation bei Erwachsenen*, PID‐5‐BF= Personality Inventory for DSM‐5‐Brief Form, RCI= Reliable Change Index, SIPP‐SF= Severity Indices of Personality Problems ‐ Short Form.

^a^ Correlation is significant at the 0.05 level (two‐tailed).

^b^ Percentage reliable improvement/percentage no reliable change/percentage deterioration.

Paired samples *t*‐tests were conducted to compare the means of the maladaptive personality traits as measured with the PID‐5‐BF‐scales at T1 and T3. The Shapiro‐Wilk tests indicated a violation of the assumption of normality for the distributions of the data for both the subscales Antagonism and Disinhibition. Therefore, for these subscales, Wilcoxon signed rank tests were performed. A statistically significant decrease was found for the total score of PID‐5‐BF (*t*(35) = 3.359, *p* = 0.002), Disinhibition (*Z* = − 2.330, *p* = 0.020), and Negative Affectivity (*t*(35) = 3.274, *p* = 0.002), with medium ES. The statistical tests comparing the scores on T1 and T3 of the remaining subscales did not reach the level of significance.

Symptom measures (i.e., BEST and BSI) demonstrated the highest percentages of reliable improvement during STEPPS. Nearly half of the participants (i.e., 41.9%) showed clear improvement in BPD‐symptoms and more than half of the participants (i.e., 57.9%) improved in symptomatic distress. Reliable improvement in emotion regulation strategies was limited; 81.1%–89.2% of the participants showed no reliable change. Similarly, most participants had no reliable change in personality functioning and maladaptive traits (i.e., 66.7%–97.2%).

## 4 Discussion

In this proof‐of‐concept‐study, we aimed to evaluate the first outcomes of the adjusted STEPPS‐OA in a sample of older adults with PDs. Previous research revealed older patients reporting less BPD‐severity, improved self‐control and improved identity integration after attending the original STEPPS program (Ekiz et al. 2023a). Results of the current STEPPS‐OA study replicated these findings. In addition to the original STEPPS, older adults attending STEPPS‐OA also reported decreased symptomatic distress and improved interpersonal functioning. This indicates that the adjustments were likely better suited to the target group. Furthermore, measurement of emotion regulation strategies revealed that adaptive strategies improved and maladaptive strategies remained unchanged. This aligns with the concept that previous behaviors or cognitions are not erased but instead, new behaviors or cognitions are created that compete with original learnings (Bouton 2004).

Although results indicate that control over emotions improved significantly, with medium to large ES, it is noteworthy that these improvements primarily seemed to occur in the first phase of the treatment, between T1 and T2. At T2, patients have received psycho‐education, the first part of the program, and have been taught three of the five emotion management skills in the previous group meetings. The improvements persisted in the last phase of the treatment, between T2 and T3. However, between T2 and T3, no further statistical significant improvement was found. This suggests that the majority of improvements were achieved during the initial phase of the program and were subsequently sustained in the latter phase. In the last phase of the treatment patients learn the remaining emotion management skills, healthy lifestyle behaviors and interpersonal effectiveness skills. Surprisingly, relational functioning also improved significantly in the first phase of the treatment, but not in the last phase. One possible explanation is that improvements in emotion regulation skills may already have a positive effect on relational functioning. Additionally, engaging the patient's support system from the outset may yield improved interpersonal effectiveness in these relationships. Another possible explanation for the early improvement is that these changes indicate a placebo response, an often underestimated factor in psychotherapy (Enck and Zipfel 2019). Factors like treatment expectancy and induced hope may have contributed to observed changes. That being said, early improvements in psychotherapy are consistent with findings from two studies investigating outcome trajectories in Dialectical Behavior Therapy (DBT) for BPD in younger adults (Yin et al. 2022; McMain et al. 2018). In these studies, patients showed a rapid decline in negative behaviors at the beginning of treatment, with the largest group of patients demonstrating the most changes early on in treatment. Future research should investigate the optimal dose‐effect relationship for STEPPS‐OA, considering both placebo‐related factors and insights from the DBT outcome studies.

Another important finding was that STEPPS‐OA was associated with a decrease of maladaptive personality traits (as measured with PID‐5‐BF), such as negative affectivity and disinhibition, and an increase of adaptive personality functioning (as measured with SIPP‐SF).

It is important to note that personality functioning differs from maladaptive traits in that it provides a broader and more general view of personality pathology (Debast et al. 2018). While maladaptive traits focus on specific problematic characteristics of PDs, personality functioning offers a more comprehensive picture of overall personality pathology.

Despite the promising effect sizes on several outcomes, data on reliable change demonstrated that reliable improvement was greatest for BPD‐symptoms and symptomatic distress, whereas predominantly no reliable change was found for emotion regulation and personality measures. Importantly, used symptom questionnaires explicitly assess symptoms within the past 7 days, whereas other used questionnaires capture more stable personality traits and behaviors. This difference may account for rapid improvements in symptoms, with no current reliable changes in personality measures.

In current sample, the drop‐out rate was 26.9%. A major reason for discontinuation was the presence or emergence of significant somatic health issues affecting patients participation. The drop‐out rate in the present study is lower than the mean drop‐out rate found in younger adults participating in STEPPS studies, that is, 42% (Ekiz et al. 2022). The drop‐out rate of current study seems to be higher than the attrition rate of 23.1% in the STEPPS pilot study (Ekiz et al. 2023a). However, after correcting for the different calculation methods used in these studies, the pilot study yields a rate of 27.3% (with a total of 33 participants, 24 completers and nine non‐completers), which is comparable to the rate in the current STEPPS‐OA study.

The results of the present study should also be seen in light of some limitations and strengths. Limitations of this study include the absence of a control group, which restricts the ability to draw definitive conclusions about the specific effects attributable to the intervention. Additionally, the absence of follow‐up data precludes assessing whether the achieved results are sustained over time. This proof‐of‐concept study was designed as an intermediate step to gain insights before proceeding with a larger trial with follow‐up data. Another limitation is the use of measurements not validated for older adults (i.e., BEST, FEEL‐E) or with suboptimal age‐neutrality (i.e., PID‐5‐BD). Furthermore, the present study lacks a qualitative description of experiences of patients attending STEPPS‐OA and an evaluation of the treatment program by the therapists. Strengths of our study include the development and implementation of a modified program, informed by expert's recommendations, which in its original format showed promising results for older adults with PD. Results indicate that the adjustments made in STEPPS‐OA are promising and support the proof‐of‐concept of the intervention. This study is particularly important because few studies have been conducted into (tailored) treatment options for older adults with BPD (Videler et al. 2019), which may result in them being under‐treated.

Based on the promising outcomes observed in this study evaluating STEPPS‐OA, the next step could be conducting a larger (randomized controlled) trial with follow‐up data. This would be beneficial in minimizing bias and allowing more reliable and valid conclusions about the effectiveness of STEPPS‐OA.

## 5 Conclusion

This proof‐of‐concept study indicates that STEPPS‐OA is promising treatment option for older adults with emotional intensity difficulties and PDs, replicating earlier findings of the original treatment program and extending these with additional significant results. STEPPS‐OA is associated with decreased BPD‐severity, psychological distress and maladaptive personality traits (i.e., negative affectivity and disinhibition), and improved adaptive emotion regulation strategies and personality functioning.

## Conflicts of Interest

The authors declare no conflicts of interest.

## Data Availability

The data that support the findings of this study are available from the corresponding author upon reasonable request.
